# Supplementary material for: Secretin Receptor as a Target in Gastrointestinal Cancer: Expression Analysis and Ligand Development
Source: Biomedicines. 2022 Feb 24;10(3):536. doi: 10.3390/biomedicines10030536 (PMC8944975; doi:10.3390/biomedicines10030536)
Supplement: Supplementary file 1 [file biomedicines-10-00536-s001.zip › biomedicines-1600036-supplementary.pdf]

Supplement

Antibody validation

A commercially available SCTR antibody (HPA007269, Sigma-Aldrich) was validated for specificity and subcellular staining. An epitope tag antibody (TAG) detected expression of SCTR equipped with this epitope tag and of the related glucagon-like receptor 2 (GLP-2R), respectively, in lysates in a Western blot. The SCTR antibody, however, detected only the expressed SCTR-TAG protein, proving specificity of the antibody for SCTR (Supplementary figure 1A). Likewise, U2OS cells stably expressing either SCTR or GLP-2R were stained with the SCTR antibody in immunofluorescence. Applying both permeabilizing and non-permeabilizing conditions, the SCTR antibody only yielded a signal in SCTR-expressing, not GLP-2R-expressing cells, confirming its high specificity. In addition, the localization of the SCTR staining at the plasma membrane was found to be consistent with the detection of a heptahelical receptor like SCTR (Supplementary figure 1B).

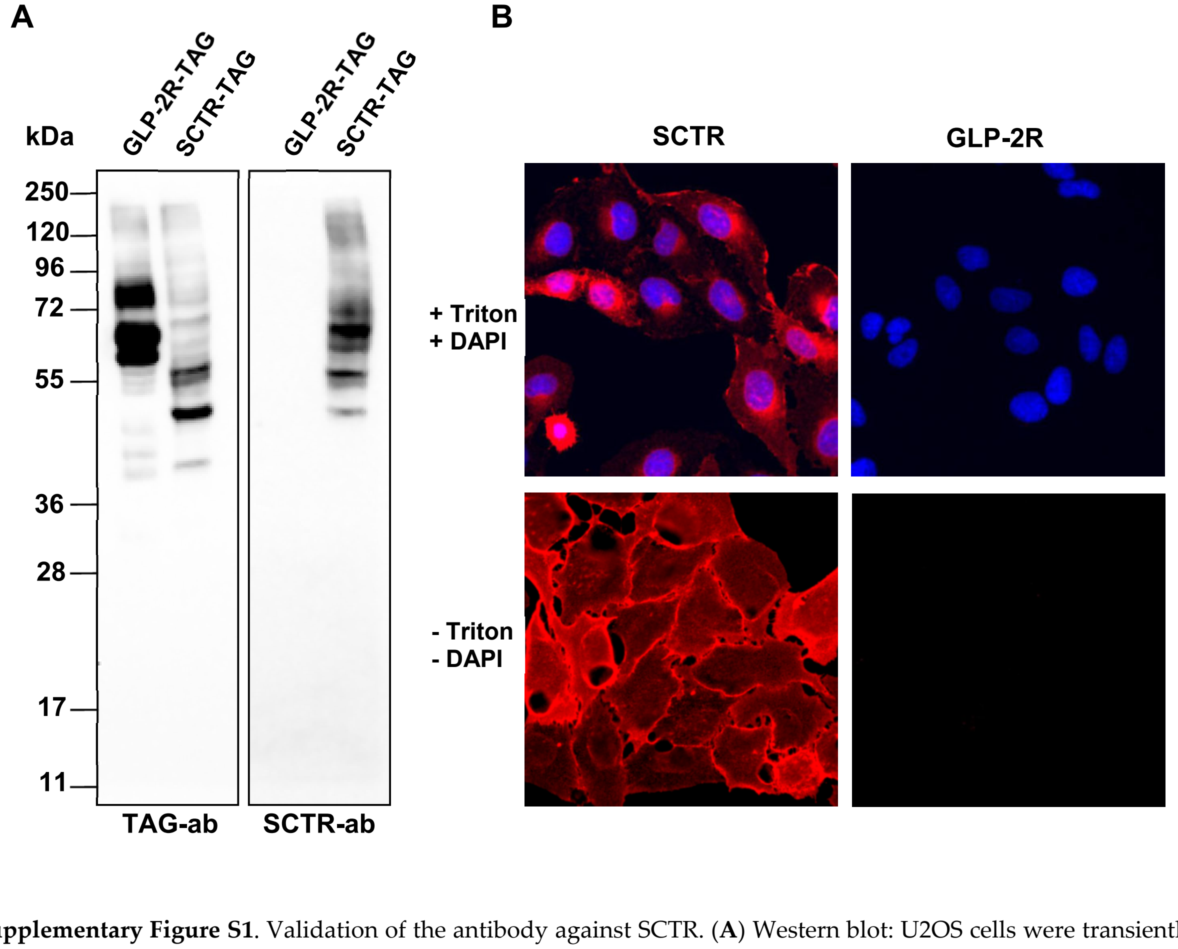

Supplementary Figure S1. Validation of the antibody against SCTR. (**A**) Western blot: U2OS cells were transiently transfected with a plasmid encoding GLP-2R equipped with an epitope tag (GLP-2R-TAG) or with a plasmid encoding SCTR with the same tag (SCTR-TAG), and lysed 24 h later. Lysates were separated by SDS-PAGE and blotted onto a nitrocellulose membrane. The left part of the membrane was incubated with an antibody against the epitope tag (TAG-ab), the right part of the membrane with the antibody against SCTR (SCTR-ab, 0.16 µg/ml, Sigma-Aldrich). Detection was performed using a POD-coupled goat anti-mouse or goat anti-rabbit antibody and SuperSignal West Dura development substrate (PIERCE). (**B**) Immunofluorescence: U2OS cells stably expressing SCTR and GLP-2R, respectively, were fixed and partially permeabilized with 0.1% Triton-X100. Cells were stained with the antibody against SCTR (0.4 µg/ml, Sigma-Aldrich) and a Cy3-labeled goat anti-rabbit antibody. The nuclei of the permeabilized cells were additionally stained with DAPI. Images were acquired on the InCell Analyzer 1000 (GE Healthcare).

High-magnification images of SCTR immunohistochemistry – Esophageal Cancer

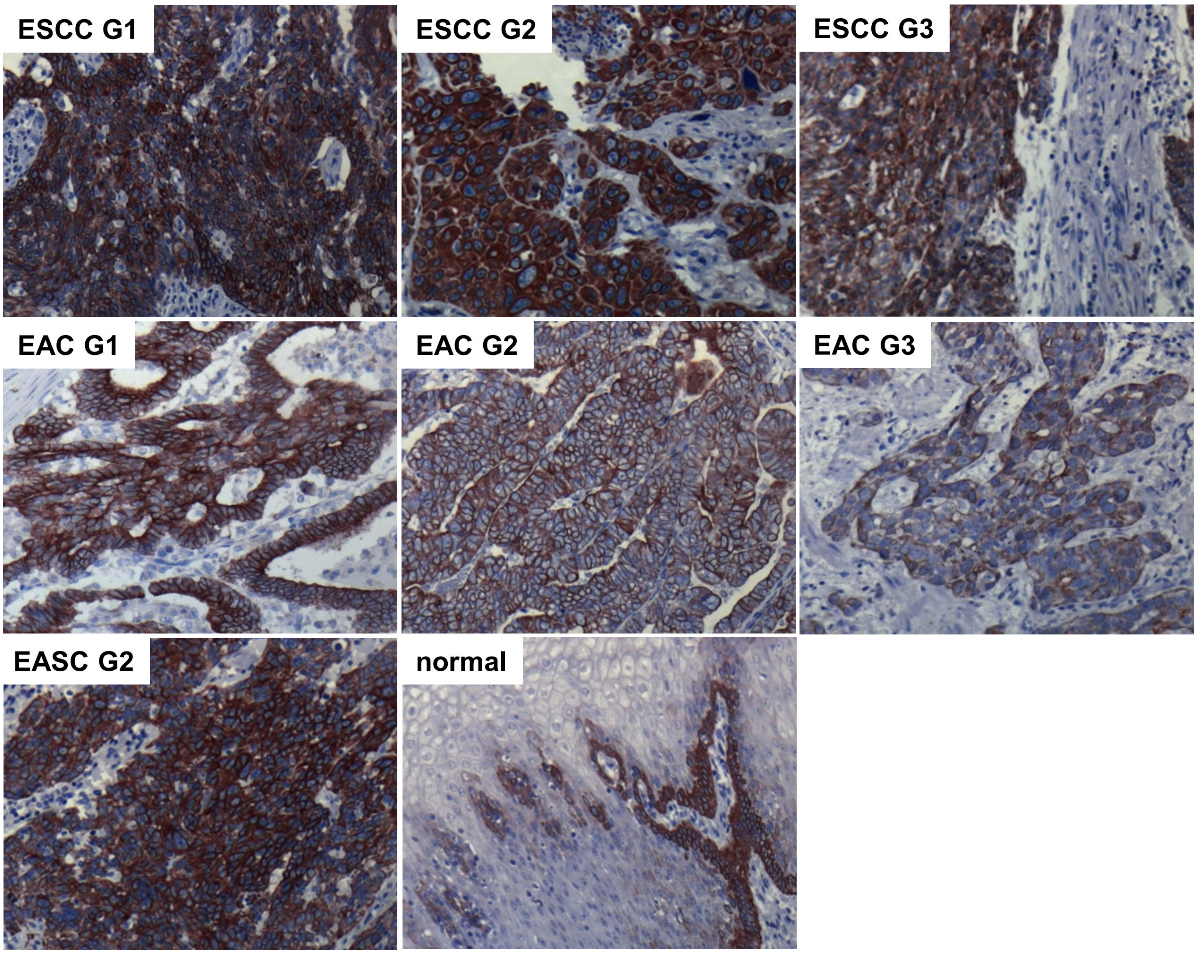

Supplementary Figure S2. Immunohistochemical detection and quantification of SCTR in esophageal cancer of various histopathological grades (G1, G2, G3) and in normal human esophagus. Representative SCTR immunohistochemistry stainings are presented for ESCC, EAC, EASC and non-cancerous esophagus tissue (normal). In addition, a quantification of the staining (IHC score) for n=30 normal tissue samples (N) as well as for the three tumor entities ESCC (n=101), EAC (n=44) and EASC (n=6) is presented. Microscopic images (same samples as in Fig. 1) were taken using a 20x objective.

High-magnification images of SCTR immunohistochemistry – Pancreatic Cancer

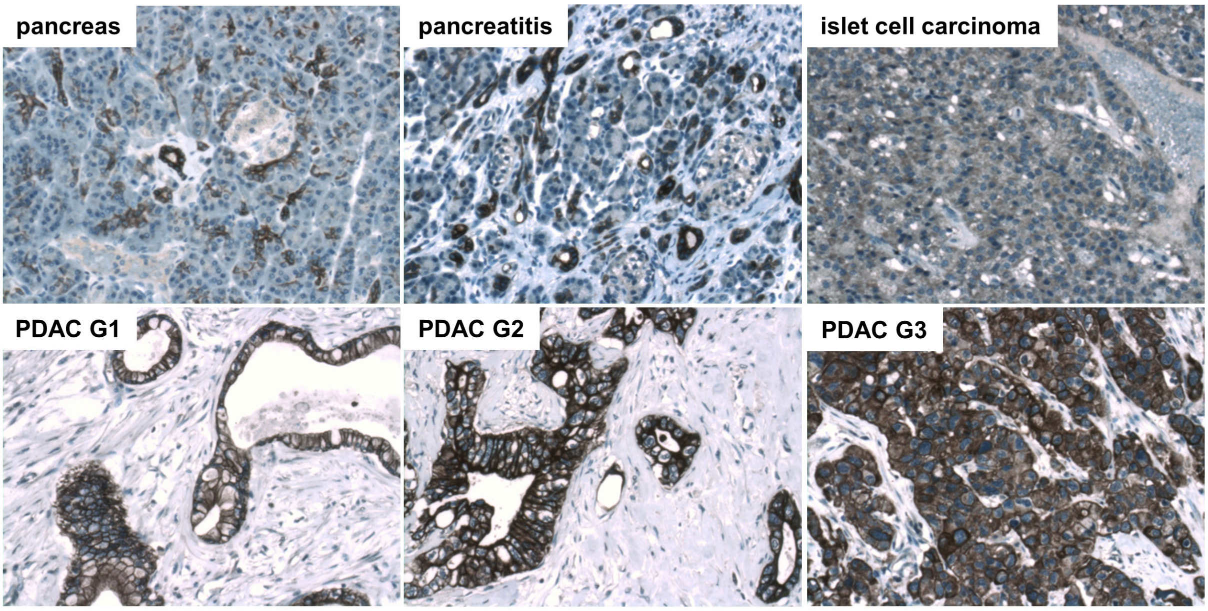

Supplementary Figure S3. Immunohistochemical detection of SCTR in human normal pancreas, chronic pancreatitis and islet cell carcinoma as well as in PDAC of histopathological grades G1, G2 and G3. Representative SCTR immunohistochemistry microphotographs are presented. Microscopic images (same samples as in Fig. 2) were taken using a 20x objective.

SCTR IHC score for different histological gradings

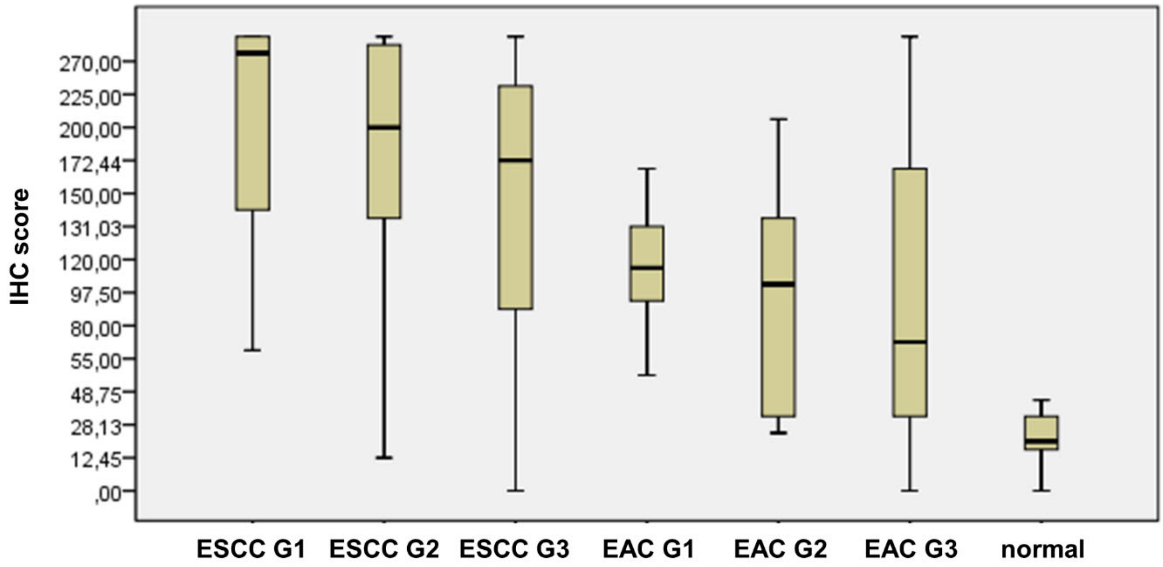

Supplementary Figure S4. Boxplot of the IHC score of SCTR antibody staining in normal and cancerous esophageal samples. ESCC and EAC IHC scores were plotted according to the histopathological grading (G1, G2 or G3) of the tumors. For comparison, the IHC score for normal esophageal tissue is shown.

SCTR staining in normal tissues

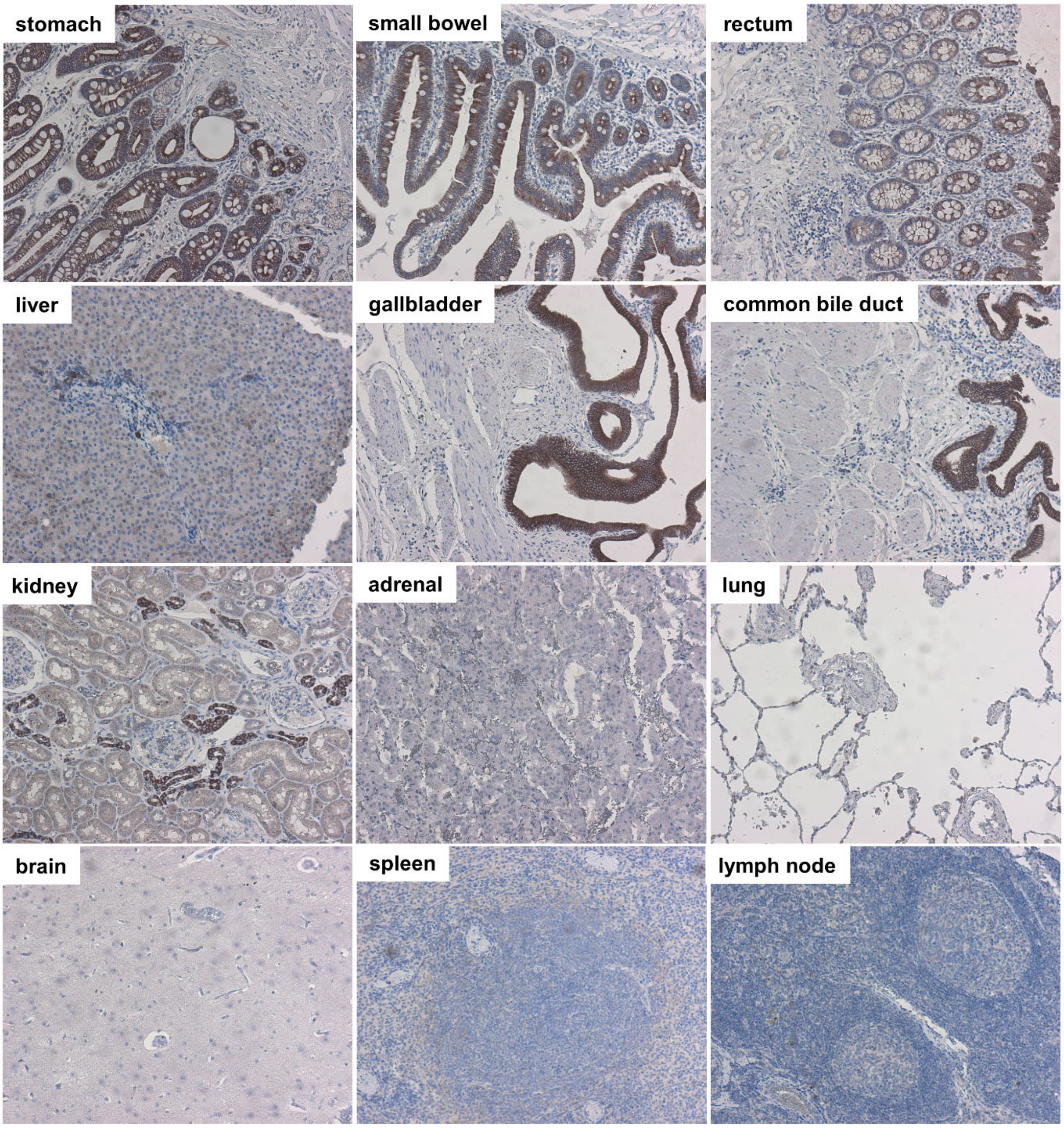

Supplementary Figure S5. SCTR immunohistochemistry of normal tissues. As for esophageal and pancreatic normal and pathological samples, a number of normal tissues were stained for SCTR, too. These included samples from stomach, small bowel, rectum, liver, gallbladder, common bile duct, kidney, adrenal, lung, brain, spleen and lymph node, each n=1. Microscopic images were taken using a 10x objective.

Supplementary Table S1

Pharmacological properties of wild-type secretin and secretin variants, part 1.

peptide	sequence																											β-arrestin2-GFP agonism				cAMP agonism	
																												potency	SD	efficacy	SD	potency	SD
	1	2	3	4	5	6	7	8	9	10	11	12	13	14	15	16	17	18	19	20	21	22	23	24	25	26	27	[nM]	[nM]	[%Sec]	[%Sec]	[nM]	[nM]
1	H	S	D	G	T	F	T	S	E	L	S	R	L	R	E	G	A	R	L	Q	R	L	L	Q	G	L	V	3,26	0,80	100,00			
2	A	S	D	G	T	F	T	S	E	L	S	R	L	R	E	G	A	R	L	Q	R	L	L	Q	G	L	V	233,86	119,49	89,10	4,71		
3	H	A	D	G	T	F	T	S	E	L	S	R	L	R	E	G	A	R	L	Q	R	L	L	Q	G	L	V	9,21	6,84	102,60	4,78		
4	H	S	A	G	T	F	T	S	E	L	S	R	L	R	E	G	A	R	L	Q	R	L	L	Q	G	L	V	1.577,92	1.386,13	65,88	24,59		
5	H	S	D	A	T	F	T	S	E	L	S	R	L	R	E	G	A	R	L	Q	R	L	L	Q	G	L	V	14,01	7,37	87,17	4,35		
6	H	S	D	G	A	F	T	S	E	L	S	R	L	R	E	G	A	R	L	Q	R	L	L	Q	G	L	V	6,99	4,31	104,45	8,49		
7	H	S	D	G	T	A	T	S	E	L	S	R	L	R	E	G	A	R	L	Q	R	L	L	Q	G	L	V	155,16	30,79	61,83	4,92		
8	H	S	D	G	T	F	A	S	E	L	S	R	L	R	E	G	A	R	L	Q	R	L	L	Q	G	L	V	131,37	48,42	83,88	12,51		
9	H	S	D	G	T	F	T	A	E	L	S	R	L	R	E	G	A	R	L	Q	R	L	L	Q	G	L	V	24,29	12,76	88,08	10,83		
10	H	S	D	G	T	F	T	S	A	L	S	R	L	R	E	G	A	R	L	Q	R	L	L	Q	G	L	V	10,87	3,36	100,17	11,42		
11	H	S	D	G	T	F	T	S	E	A	S	R	L	R	E	G	A	R	L	Q	R	L	L	Q	G	L	V	120,52	23,18	96,50	9,58		
12	H	S	D	G	T	F	T	S	E	L	A	R	L	R	E	G	A	R	L	Q	R	L	L	Q	G	L	V	5,76	1,89	101,60	10,47		
13	H	S	D	G	T	F	T	S	E	L	S	A	L	R	E	G	A	R	L	Q	R	L	L	Q	G	L	V	6,06	2,04	100,37	18,43		
14	H	S	D	G	T	F	T	S	E	L	S	R	A	R	E	G	A	R	L	Q	R	L	L	Q	G	L	V	2,71	1,46	99,50	12,76		
15	H	S	D	G	T	F	T	S	E	L	S	R	L	A	E	G	A	R	L	Q	R	L	L	Q	G	L	V	13,38	2,04	106,30	3,58		
16	H	S	D	G	T	F	T	S	E	L	S	R	L	R	A	G	A	R	L	Q	R	L	L	Q	G	L	V	16,41	3,16	105,27	7,71		
17	H	S	D	G	T	F	T	S	E	L	S	R	L	R	E	A	A	R	L	Q	R	L	L	Q	G	L	V	2,63	0,90	108,87	7,40		
18	H	S	D	G	T	F	T	S	E	L	S	R	L	R	E	G	A	A	L	Q	R	L	L	Q	G	L	V	5,37	1,66	110,97	5,59		
19	H	S	D	G	T	F	T	S	E	L	S	R	L	R	E	G	A	R	A	Q	R	L	L	Q	G	L	V	62,53	8,55	107,03	4,54		
20	H	S	D	G	T	F	T	S	E	L	S	R	L	R	E	G	A	R	L	A	R	L	L	Q	G	L	V	4,60	3,31	108,60	13,30		
21	H	S	D	G	T	F	T	S	E	L	S	R	L	R	E	G	A	R	L	Q	A	L	L	Q	G	L	V	6,35	1,75	113,40	5,01		
22	H	S	D	G	T	F	T	S	E	L	S	R	L	R	E	G	A	R	L	Q	R	A	L	Q	G	L	V	23,86	4,34	110,87	4,31		
23	H	S	D	G	T	F	T	S	E	L	S	R	L	R	E	G	A	R	L	Q	R	L	A	Q	G	L	V	20,76	3,75	108,53	4,58		
24	H	S	D	G	T	F	T	S	E	L	S	R	L	R	E	G	A	R	L	Q	R	L	L	A	G	L	V	4,71	2,01	111,70	6,77		
25	H	S	D	G	T	F	T	S	E	L	S	R	L	R	E	G	A	R	L	Q	R	L	L	Q	A	L	V	4,12	0,49	119,50	9,15		
26	H	S	D	G	T	F	T	S	E	L	S	R	L	R	E	G	A	R	L	Q	R	L	L	Q	G	A	V	14,96	2,19	112,30	4,00		
27	H	S	D	G	T	F	T	S	E	L	S	R	L	R	E	G	A	R	L	Q	R	L	L	Q	G	L	A	7,20	2,60	114,57	7,50		
28	R	S	D	G	T	F	T	S	E	L	S	R	L	R	E	G	A	R	L	Q	R	L	L	Q	G	L	V	475,36	166,97	66,47	6,36		
29	E	S	D	G	T	F	T	S	E	L	S	R	L	R	E	G	A	R	L	Q	R	L	L	Q	G	L	V	671,37	180,62	63,70	3,24		
30	F	S	D	G	T	F	T	S	E	L	S	R	L	R	E	G	A	R	L	Q	R	L	L	Q	G	L	V	97,34	22,95	67,60	5,09		
31	H	W	D	G	T	F	T	S	E	L	S	R	L	R	E	G	A	R	L	Q	R	L	L	Q	G	L	V	41,63	24,81	20,17	4,55		
32	H	R	D	G	T	F	T	S	E	L	S	R	L	R	E	G	A	R	L	Q	R	L	L	Q	G	L	V	-	-	-	-		
33	H	D	D	G	T	F	T	S	E	L	S	R	L	R	E	G	A	R	L	Q	R	L	L	Q	G	L	V	-	-	-	-		
34	H	S	R	G	T	F	T	S	E	L	S	R	L	R	E	G	A	R	L	Q	R	L	L	Q	G	L	V	-	-	-	-		
35	H	S	N	G	T	F	T	S	E	L	S	R	L	R	E	G	A	R	L	Q	R	L	L	Q	G	L	V	315,45	123,95	105,20	9,30		
36	H	S	W	G	T	F	T	S	E	L	S	R	L	R	E	G	A	R	L	Q	R	L	L	Q	G	L	V	-	-	-	-		
37	H	S	D	R	T	F	T	S	E	L	S	R	L	R	E	G	A	R	L	Q	R	L	L	Q	G	L	V	-	-	-	-		
38	H	S	D	E	T	F	T	S	E	L	S	R	L	R	E	G	A	R	L	Q	R	L	L	Q	G	L	V	-	-	-	-		
39	H	S	D	I	T	F	T	S	E	L	S	R	L	R	E	G	A	R	L	Q	R	L	L	Q	G	L	V	-	-	-	-		
40	H	S	D	G	R	F	T	S	E	L	S	R	L	R	E	G	A	R	L	Q	R	L	L	Q	G	L	V	4,16	0,70	106,47	4,48		
41	H	S	D	G	D	F	T	S	E	L	S	R	L	R	E	G	A	R	L	Q	R	L	L	Q	G	L	V	4,10	0,84	103,67	6,37		
42	H	S	D	G	C	F	T	S	E	L	S	R	L	R	E	G	A	R	L	Q	R	L	L	Q	G	L	V	9,66	1,70	107,17	7,43		
43	H	S	D	G	T	R	T	S	E	L	S	R	L	R	E	G	A	R	L	Q	R	L	L	Q	G	L	V	-	-	-	-		
44	H	S	D	G	T	D	T	S	E	L	S	R	L	R	E	G	A	R	L	Q	R	L	L	Q	G	L	V	-	-	-	-		
45	H	S	D	G	T	V	T	S	E	L	S	R	L	R	E	G	A	R	L	Q	R	L	L	Q	G	L	V	61,03	22,75	109,27	2,75		
46	H	S	D	G	T	F	R	S	E	L	S	R	L	R	E	G	A	R	L	Q	R	L	L	Q	G	L	V	-	-	-	-		
47	H	S	D	G	T	F	D	S	E	L	S	R	L	R	E	G	A	R	L	Q	R	L	L	Q	G	L	V	-	-	-	-		
48	H	S	D	G	T	F	C	S	E	L	S	R	L	R	E	G	A	R	L	Q	R	L	L	Q	G	L	V	540,62	288,00	102,57	11,14		
49	H	S	D	G	T	F	T	R	E	L	S	R	L	R	E	G	A	R	L	Q	R	L	L	Q	G	L	V	-	-	-	-		
50	H	S	D	G	T	F	T	D	E	L	S	R	L	R	E	G	A	R	L	Q	R	L	L	Q	G	L	V	38,99	6,87	86,87	3,95		
51	H	S	D	G	T	F	T	W	E	L	S	R	L	R	E	G	A	R	L	Q	R	L	L	Q	G	L	V	-	-	-	-		
52	H	S	D	G	T	F	T	S	R	L	S	R	L	R	E	G	A	R	L	Q	R	L	L	Q	G	L	V	18,64	2,30	94,23	11,42		
53	H	S	D	G	T	F	T	S	L	L	S	R	L	R	E	G	A	R	L	Q	R	L	L	Q	G	L	V	15,98	5,09	106,43	7,73		
54	H	S	D	G	T	F	T	S	Y	L	S	R	L	R	E	G	A	R	L	Q	R	L	L	Q	G	L	V	11,75	1,29	103,23	7,20		
55	H	S	D	G	T	F	T	S	E	R	S	R	L	R	E	G	A	R	L	Q	R	L	L	Q	G	L	V	154,10	58,03	86,62	7,70		
56	H	S	D	G	T	F	T	S	E	D	S	R	L	R	E	G	A	R	L	Q	R	L	L	Q	G	L	V	869,29	640,61	62,80	12,45		
57	H	S	D	G	T	F	T	S	E	N	S	R	L	R	E	G	A	R	L	Q	R	L	L	Q	G	L	V	213,65	121,19	83,67	11,03		
58	H	S	D	G	T	F	T	S	E	L	S	R	L	R	E	G	A	R	L	Q	R	L	L	R	G	L	V	2,95	0,65	113,73	3,59		
59	H	S	D	G	T	F	T	S	E	L	S	R	L	R	E	G	A	R	L	Q	R	L	L	E	G	L	V	3,47	0,19	108,90	6,08		
60	H	S	D	G	T	F	T	S	E	L	S	R	L	R	E	G	A	R	L	Q	R	L	L	L	G	L	V	4,14	1,80	116,10	3,22		
61	H	S	D	G	T	F	T	S	E	L	S	R	L	R	E	G	A	R	L	Q	R	L	L	Q	R	L	V	5,25	2,09	112,00	2,00		
62	H	S	D	G	T	F	T	S	E	L	S	R	L	R	E	G	A	R	L	Q	R	L	L	Q	E	L	V	4,32	2,03	111,30	4,24		
63	H	S	D	G	T	F	T	S	E	L	S	R	L	R	E	G	A	R	L	Q	R	L	L	Q	I	L	V	7,44	4,42	106,87	0,85		
64	H	S	D	G	T	F	T	S	E	L	S	R	L	R	E	G	A	R	L	Q	R	L	L	Q	C	L	V	20,93	10,02	110,93	0,75		
65	H	S	D	G	T	F	T	S	E	L	S	R	L	R	E	G	A	R	L	Q	R	L	L	Q	G	R	V	9,06	3,94	105,47	1,21		
66	H	S	D	G	T	F	T	S	E	L	S	R	L	R	E	G	A	R	L	Q	R	L	L	Q	G	D	V	15,32	3,85	105,00	2,78		
67	H	S	D	G	T	F	T	S	E	L	S	R	L	R	E	G	A	R	L	Q	R	L	L	Q	G	F	V	12,00	4,16	103,53	2,33		

Supplementary Table S2

Pharmacological properties of secretin variants, part 2.

peptide	sequence 1	2	3	4	5	6	7	8	9	10	11	12	13	14	15	16	17	18	19	20	21	22	23	24	25	26	27	β-arrestin2-GFP agonism potency [nM]	SD [nM]	efficacy [% Sec]	SD [% Sec]	cAMP agonism potency [nM]	SD [nM]
68	H	S	D	G	T	F	T	S	E	L	S	R	L	K	E	G	A	R	L	Q	R	L	L	Q	G	L	V	9,53	1,58	114,30	7,33		
69	H	S	D	G	T	F	T	S	E	L	S	R	L	R	D	G	A	R	L	Q	R	L	L	Q	G	L	V	4,52	1,61	111,23	6,90		
70	H	S	D	G	T	F	T	S	E	L	S	R	L	R	E	G	E	R	L	Q	R	L	L	Q	G	L	V	2,26	0,71	104,77	2,89		
71	H	S	D	G	T	F	T	S	E	L	S	R	L	R	E	G	A	R	L	E	R	L	L	Q	G	L	V	2,28	0,54	100,73	2,80		
72	H	S	D	G	T	F	T	S	E	L	S	R	L	R	E	L	A	R	L	Q	R	L	L	Q	G	L	V	3,32	0,60	104,60	4,52		
73	P	S	D	G	T	F	T	S	E	L	S	R	L	R	E	G	A	R	L	Q	R	L	L	Q	G	L	V	39,08	12,83	96,57	6,31		
74	H	P	D	G	T	F	T	S	E	L	S	R	L	R	E	G	A	R	L	Q	R	L	L	Q	G	L	V	3,37	1,99	106,23	2,44		
75	H	S	P	G	T	F	T	S	E	L	S	R	L	R	E	G	A	R	L	Q	R	L	L	Q	G	L	V	-	-	-	-		
76	H	S	D	P	T	F	T	S	E	L	S	R	L	R	E	G	A	R	L	Q	R	L	L	Q	G	L	V	-	-	-	-		
77	H	S	D	G	P	F	T	S	E	L	S	R	L	R	E	G	A	R	L	Q	R	L	L	Q	G	L	V	-	-	-	-		
78	H	S	D	G	T	P	T	S	E	L	S	R	L	R	E	G	A	R	L	Q	R	L	L	Q	G	L	V	-	-	-	-		
79	H	S	D	G	T	F	P	S	E	L	S	R	L	R	E	G	A	R	L	Q	R	L	L	Q	G	L	V	-	-	-	-		
80	H	S	D	G	T	F	T	P	E	L	S	R	L	R	E	G	A	R	L	Q	R	L	L	Q	G	L	V	-	-	-	-		
81	H	S	D	G	T	F	T	S	P	L	S	R	L	R	E	G	A	R	L	Q	R	L	L	Q	G	L	V	-	-	-	-		
82	H	S	D	G	T	F	T	S	E	P	S	R	L	R	E	G	A	R	L	Q	R	L	L	Q	G	L	V	-	-	-	-		
83	H	S	D	G	T	F	T	S	E	L	S	R	L	R	E	G	A	R	L	Q	R	L	L	Q	P	L	V	22,13	10,01	103,17	4,12		
84	H	S	D	G	T	F	T	S	E	L	S	R	L	R	E	G	A	R	L	Q	R	L	L	Q	G	P	V	72,64	22,88	95,03	7,74		
85	H	S	D	G	T	F	T	S	E	L	S	R	L	R	E	G	A	R	L	Q	R	L	L	Q	G	L	P	5,19	2,62	100,47	11,91		
86	h	S	D	G	T	F	T	S	E	L	S	R	L	R	E	G	A	R	L	Q	R	L	L	Q	G	L	V	13,59	4,52	107,20	1,67		
87	H	s	D	G	T	F	T	S	E	L	S	R	L	R	E	G	A	R	L	Q	R	L	L	Q	G	L	V	45,06	21,00	91,57	6,20		
88	H	S	d	G	T	F	T	S	E	L	S	R	L	R	E	G	A	R	L	Q	R	L	L	Q	G	L	V	135,95	67,03	89,07	1,87		
89	H	S	D	G	t	F	T	S	E	L	S	R	L	R	E	G	A	R	L	Q	R	L	L	Q	G	L	V	308,43	31,06	102,97	5,57		
90	H	S	D	G	T	f	T	S	E	L	S	R	L	R	E	G	A	R	L	Q	R	L	L	Q	G	L	V	-	-	-	-		
91	H	S	D	G	T	F	t	S	E	L	S	R	L	R	E	G	A	R	L	Q	R	L	L	Q	G	L	V	1.537,76	534,38	98,33	5,69		
92	H	S	D	G	T	F	T	s	E	L	S	R	L	R	E	G	A	R	L	Q	R	L	L	Q	G	L	V	1.873,16	538,08	90,37	6,84		
93	H	S	D	G	T	F	T	S	e	L	S	R	L	R	E	G	A	R	L	Q	R	L	L	Q	G	L	V	20,35	6,53	100,40	5,89		
94	H	S	D	G	T	F	T	S	E	l	S	R	L	R	E	G	A	R	L	Q	R	L	L	Q	G	L	V	20,99	5,47	94,17	6,05		
95	H	S	D	G	T	F	T	S	E	L	S	R	L	R	E	G	A	R	L	Q	R	L	L	Q	G	l	V	8,48	3,66	101,90	4,71		
96	H	S	D	G	T	F	T	S	E	L	S	R	L	R	E	G	A	R	L	Q	R	L	L	Q	G	V	v	4,73	2,07	99,97	3,64		
97	H	S	D	G	T	F	T	S	E	L	S	R	L	R	E	G	A	R	L	Q	R	L	L	Q	G	L		8,80	2,92	109,53	9,99	0,51	0,17
98	H	S	D	G	T	F	T	S	E	L	S	R	L	R	E	G	A	R	L	Q	R	L	L	Q	G			26,54	4,55	104,47	14,11	1,55	0,26
99	H	S	D	G	T	F	T	S	E	L	S	R	L	R	E	G	A	R	L	Q	R	L	L	Q				35,92	7,43	103,60	9,27	2,09	0,43
100	H	S	D	G	T	F	T	S	E	L	S	R	L	R	E	G	A	R	L	Q	R	L	L					130,92	21,82	92,23	5,84	7,63	1,27
101	H	S	D	G	T	F	T	S	E	L	S	R	L	R	E	G	A	R	L	Q	R	L										57,02	3,56
102	H	S	D	G	T	F	T	S	E	L	S	R	L	R	E	G	A	R	L	Q	R							-	-	-	-	585,95	531,03
103	H	S	D	G	T	F	T	S	E	L	S	R	L	R	E	G	A	R	L	Q								-	-	-	-	874,80	718,23
104	H	S	D	G	T	F	T	S	E	L	S	R	L	R	E	G	A	R	L									-	-	-	-	2.213,08	1.474,81
105	H	S	D	G	T	F	T	S	E	L	S	R	L	R	E	G	A	R										-	-	-	-	4.710,28	3.157,82
106	H	S	D	G	T	F	T	S	E	L	S	R	L	R	E	G	A											-	-	-	-	9.433,15	7.095,81
107	H	S	D	G	T	F	T	S	E	L	S	R	L	R	E	G												-	-	-	-	20.152,03	14.941,03
108	H	S	D	G	T	F	T	S	E	L	S	R	L	R	E													-	-	-	-	-	-
109	H	S	D	G	T	F	T	S	E	L	S	R	L	R														-	-	-	-	6.025,18	5.317,17
110	H	S	D	G	T	F	T	S	E	L	S	R	L															-	-	-	-	11.619,34	
111	H	S	D	G	T	F	T	S	E	L	S	R																-	-	-	-	-	-
112	H	S	D	G	T	F	T	S	E	L	S																	-	-	-	-	-	-
113	H	S	D	G	T	F	T	S	E	L																		-	-	-	-	-	-
114	H	S	D	G	T	F	T	S	E																			-	-	-	-	12.600,00	
115		S	D	G	T	F	T	S	E	L	S	R	L	R	E	G	A	R	L	Q	R	L	L	Q	G	L	V	-	-	-	-	21,96	
116			D	G	T	F	T	S	E	L	S	R	L	R	E	G	A	R	L	Q	R	L	L	Q	G	L	V	-	-	-	-	148,71	
117				G	T	F	T	S	E	L	S	R	L	R	E	G	A	R	L	Q	R	L	L	Q	G	L	V	-	-	-	-	165,99	
118					T	F	T	S	E	L	S	R	L	R	E	G	A	R	L	Q	R	L	L	Q	G	L	V	-	-	-	-	578,32	359,87
119						F	T	S	E	L	S	R	L	R	E	G	A	R	L	Q	R	L	L	Q	G	L	V	-	-	-	-	1.112,83	671,19
120							T	S	E	L	S	R	L	R	E	G	A	R	L	Q	R	L	L	Q	G	L	V	-	-	-	-	3.366,30	1.570,29
121								S	E	L	S	R	L	R	E	G	A	R	L	Q	R	L	L	Q	G	L	V	-	-	-	-	-	-
122									E	L	S	R	L	R	E	G	A	R	L	Q	R	L	L	Q	G	L	V	-	-	-	-	5.852,93	762,96
123										L	S	R	L	R	E	G	A	R	L	Q	R	L	L	Q	G	L	V	-	-	-	-	9.495,41	5.881,85
